# Professionals’ perception of intimate partner violence in young people: a qualitative study in northern Spain

**DOI:** 10.1186/s12978-017-0348-8

**Published:** 2017-07-20

**Authors:** Amaia Maquibar, Carmen Vives-Cases, Anna-Karin Hurtig, Isabel Goicolea

**Affiliations:** 1 Department of Nursing I, Faculty of Medicine and Nursing, University of the Basque Country UPV/EHU, Leioa, Bizkaia Spain; 2 Public Health Research Group, Alicante University, Alicante, Spain; 3 Department of Community Nursing, Preventive Medicine and Public Health and History of Science, Alicante University, Alicante, Spain; 4 CIBER of Epidemiology and Public Health (CIBERESP), Madrid, Spain; 5 Epidemiology and Global Health Unit, Department of Public Health and Clinical Medicine, Umea University, SE-90187 Umea, Sweden

**Keywords:** Intimate partner violence, Young people, Professional role, Qualitative research

## Abstract

**Background:**

Intimate partner violence (IPV) is a public health problem with devastating effects on young women’s health. These negative effects increase when the exposure to IPV lasts for a long time and exposure at an early age increases the risk of adult IPV. Despite efforts made in the last few decades, data show little progress has been made towards its reduction. Thus, the aim of the study reported here is to explore professionals’ perceptions regarding intimate partner violence (IPV) among young people, focusing on the characteristics of the phenomenon and their perceptions about existing programmes and campaigns aimed at addressing it.

**Methods:**

Twelve professionals from education, health and municipal social services were interviewed. All but one of the interviews were recorded and transcribed verbatim. Data were analysed according to the methodology of inductive thematic analysis, with the support of Atlas.ti software. The transcripts were read several times and coded line by line. Afterwards, codes were grouped into themes. The developed themes were refined into two phases with the participation of all the authors.

**Results:**

From the analysis, the following three themes were identified: “A false sense of gender equity”, “IPV among young people: subtle, daily and normalized”, and “Mass media campaigns do not fit young people’s needs”.

According to the participants, psychological abuse in the form of controlling behaviour by their partners is the most common type of IPV young women are exposed to, although exposure to other types of IPV was also acknowledged. This violence was described as something subtle, daily and normalized and, consequently, not something that is easy to recognize for the girls that are exposed to it, nor for adults working with young people.

**Conclusions:**

The study participants showed good knowledge of the characteristics IPV has among young people. This knowledge was reflected in locally implemented IPV prevention projects, which they considered successful in addressing young people’s needs. However, these interventions lacked formal evaluation, political support and continuation. The study participants did not believe that nationwide mass media campaigns realistically reflected the specific characteristics of IPV among young people. Thus, participants perceived these campaigns to be ineffective.

## Plain English summary

Violence between intimate partners (Intimate Partner Violence) is a current relevant public health problem, due to the large number of women that suffer from it and because of the negative consequences it has for their health. This study explores how professionals from education, health and municipal social services understand Intimate Partner Violence among young people as well as their opinions about current programmes and mass media campaigns aimed at preventing this type of violence. To achieve these two objectives, we interviewed 12 professionals and analysed the transcripts of what they said. We found that for the participants, psychological abuse in the form of controlling behaviour was the most common type of partner violence among young people. They described violence as something subtle, daily and normalized, and consequently girls exposed to violence might not be aware of it.

Participants perceived local prevention projects as successful in addressing young people’s needs, but these interventions lacked formal evaluation, political support and continuation. In contrast, participants did not consider that nationwide mass media campaigns reflected the specific characteristics of IPV among young people and, thus, they were perceived to be ineffective.

## Background

Intimate partner violence (IPV) *“includes physical violence, sexual violence, stalking and psychological aggression (including coercive tactics) by a current or former intimate partner (i.e., spouse, boyfriend/girlfriend, dating partner, or ongoing sexual partner)*” [1]. IPV against women has devastating effects on the health of women, including mental distress, depression, chronic pain and an overall poorer health status, to cite just a few outcomes [2, 3]. Existing studies show that IPV is a global health issue. The World Health Organization (WHO) estimates that one in three women worldwide will suffer from violence during their lifetime [4] and the results of a recent survey conducted in Europe found that one in five women had experienced physical and/or sexual violence from either a current or previous partner [5]. In addition, almost 40% of female homicides worldwide have been estimated to be committed by their partners or ex-partners [6]. In Spain, according to official data from the Ministry of Health, an average of 50 women have been killed each year by partners or ex-partners since 2003, when this registry began [7].

In the USA, the latest report from The Youth Risk Behavior Surveillance System of the Centers for Disease Control and Prevention states that 13% of women who dated someone during the last few months were exposed to physical dating violence and 14.4% were exposed to sexual dating violence [8]. Among college-aged students 19 to 25 years old, IPV rates range from 13% to 30% [9]. In Spain, according to the 2015 *Survey on Violence Against Women* conducted by the Spanish Ministry of Health, Social Services and Equity, almost 20% of women between 16 and 24 years old reported exposure to psychological control violence during their lifetime, 10.3% to physical violence and 5.7% to sexual violence [10]. The study *Gender Violence in Spanish Colleges*, conducted in six Spanish universities (including the Basque Country University) during 2006–2008, found that 65% of university students had suffered or knew about a gender-based violent act that had occurred in the university environment [9].

Just as in adulthood, IPV has a detrimental effect on young women’s health, increasing the odds of substance abuse, unhealthy weight control behaviours, sexual risk behaviours, unwanted pregnancy and suicide [11, 12]. Moreover, negative effects on health increase the longer the time the victimization lasts. Additionally, victimization during adolescence/youth increases the risk of suffering IPV during adulthood [13].

Although exposure to IPV usually begins at a young age [14], the attention paid to IPV at early age stages in international research has been for a long time considerably lower than that paid to IPV against adult women and has usually been focused exclusively on teenagers [15, 16]. During the last few decades, research in this field has markedly increased [17]. However, there is no strong evidence yet about what must be done to effectively prevent IPV. Prevention strategies to date have focused mainly on school-based programmes, with promising results in terms of changing attitudes and knowledge when intervention is comprehensive and includes communities besides the school [14, 18, 19]. Less evidence is found regarding long-term behavioural changes [20]. Systematic reviews trying to assess the effectiveness of these interventions have found important methodological weaknesses in the evaluation of the outcomes [18–20].

In addition to education, although the literature is scarce, there is some evidence which shows that policies enacted as a provision of civil protection orders for women exposed to IPV can have a positive impact in reducing the number of cases [21]. Frontline professionals play a key role in the impact of implemented policies and programmes [22]. Policymakers’ and service providers’ discourses do not merely reflect their opinions, but actually influence the services that are made available for young people and the ways they are delivered [23].

Considering the available evidence on IPV prevention, Spain, including the Basque Country, has enacted policies to address gender inequity and IPV for more than 10 years [24, 25] and has promoted school-based programmes [26]. Despite this, research shows little progress has been made and there have even been some backwards steps taken in the process of addressing IPV, as an increased tolerance towards this violence among young people [17]. Therefore, it is necessary to gain better knowledge of the characteristics of IPV among young people and to understand the role of prevention strategies currently in effect.

We conducted a study aimed at exploring professionals’ perceptions regarding intimate partner violence [IPV] among young people, focusing on the characteristics of the phenomenon and their perceptions of existing programmes and campaigns aimed at addressing it.

## Methods

### Study setting

This study is part of a larger research project on IPV and young people in Basque Country, Spain. In addition to surveying professionals from different areas, this research project will explore young people’s perspectives and attitudes towards IPV and the role of health services in IPV management.

The Basque Country is one of the 17 autonomous regions into which Spain is divided. In Spain, responsibility for implementing health, education and gender equity policies and programmes lies at the level of these autonomous regions. In the field of IPV and young people, from an institutional perspective, there are four relevant areas to consider in the Basque Country: formal education, non-formal education, health and municipal equity departments.

With respect to formal education, the Basque government is currently piloting a plan for gender violence prevention in the education sector in volunteer schools, with the aim of preventing all forms of gender-based violence. Besides formal education, all municipalities have youth departments, which provide recreational and education services to young people during weekends, holidays and on working days after school.

In the public regional health system, a group of professionals have joined together to enhance the health sector’s response to IPV. They develop awareness-raising campaigns for professionals and the general public, including some activities focused specifically on the prevention of IPV among young girls and boys.

Finally, all municipalities of the Basque Country have at least one full-time or part-time person responsible for gender equity issues. These “equity technicians” are responsible, among other tasks, for IPV prevention and also provide support and follow-up to women exposed to IPV, both in the adult and youth populations.

### Participants

For this study, we conducted in-depth interviews with 12 professionals from each of the four sectors in Basque Country involved in IPV and youth (formal education, non-formal education, health and municipal equity). Participants were identified and selected by the study investigators as being the most relevant for this study, due to their work with young people and involvement in the development and/or implementation of IPV prevention interventions. Four of the participants were “equity technicians” actively involved in the implementation of unique region-wide IPV prevention interventions among young people in the Basque Country. Two participants were civil servants from the health system involved in the working group to address IPV. Four were social educators hired by municipalities to provide comprehensive attention to young people, from academic to emotional support as well as follow-up if needed. One study participant was a university lecturer who taught a course on gender issues and IPV and another was a secondary school teacher responsible for the implementation of the gender-based violence prevention project launched by the Basque Government at her school.

### Data collection

Data collection took place from April to August 2014. Although saturation had already been achieved, during the data analysis it was considered relevant to interview a key informant from the formal education sector; thus, the last interview was conducted in May 2016. The first author (AM) conducted all individual interviews, following an open guide. Interviewees were asked about their perceptions of IPV and gender relations, measures that were being implemented that they thought were useful, and what was left to do to successfully address this issue, including coordination issues between all the actors involved.

Potential study participants were first contacted by email to explain the aim of the research study and the kind of participation required, and to ask about their willingness to take part. Once participants provided consent, they were contacted again to set a date and place for the interview. A local organization and a politician declined to take part in the study. Despite willingness to participate, it was impossible to find a suitable date for the interview with two other potential participants and we did not receive a response from another organization. Ten of the 12 interviews were held in the workplaces of the interviewees and two in coffee shops. Eleven interviews were recorded (one refused to consent to being recorded, so written notes were taken during the interview) and transcribed verbatim. The interviews lasted on average 1 h. All interviews were conducted in Spanish.

### Data analysis

Interview transcripts were entered into Atlas.ti 1.0.16 for analysis. At the beginning of each interview transcript, a brief log of the interview was written, including information about the professional background of the interviewee, the place, hour, duration, and the feelings and perceptions of the interviewer during the conversation, in order to help with the analysis process.

We analysed all the interview transcripts using the thematic analysis described by Braun and Clarke [27]. Data analysis was inductive, thus thematic construction was data-driven; no initial hypothesis guided the preliminary coding and subsequent thematic development. We adopted a constructionist perspective, as IPV is strongly rooted in societal norms and culture.

The same researcher who conducted the interviews completed the transcriptions and initial coding of the data set. One investigator performed line-by-line coding of interview transcripts, whereby codes were assigned to meaningful pieces of the text. Although the transcripts and codes were in Spanish, themes were developed in English as this was the language in which the final report was to be written. All the authors understand both languages and, thus, were able to participate in the whole analysis process. All the codes were then sorted into potential themes. Thematic maps were used at this stage to help with theme grouping and the analysis of relationships between emerging themes and codes.

The identified themes were refined using the two stages proposed by Braun and Clarke [27] with the participation of all the authors. First, all the coded extracts for each theme were read thoroughly to check coherence in the pattern that led to that theme definition. Once necessary adjustments had been made, the preliminary thematic map was confronted with the whole data set, refining themes and subthemes. A detailed analysis of each theme, including the meaning and scope, as well as relations with the other themes, was conducted and written based on the data extracts coded in each one, as suggested by Braun and Clarke [27].

### Ethics

Written informed consent was provided by all study participants and, to ensure confidentiality, all names were erased. Ethical approval for this study was granted by the Ethical Committee for Research with Human Beings of the University of the Basque Country.

## Results

From the analysis, the following three themes were identified: “A false sense of gender equity”, “IPV among young people: subtle, daily and normalized” and “Mass media campaigns do not fit young people’s needs”.

### A false sense of gender equity

One idea that emerged very strongly during the interviews was that there has been huge progress in the field of “formal equity” (laws, public discourses, access to work, etc.) but, in contrast, there have been few changes in the daily gender relations between men and women, and existing changes do not necessarily increase equity.

*“We’ve got formal equity at a legal level, but not a real equity in the cultural construction of the society we live in; so, yes, we’ve made progress because we certainly now have some laws and equity policies.”* Key Informant 4.

*“Nowadays it’s not a reality, nor is it [a reality] in the labour market; women continue to have lower salaries, [to work] at lower levels. We can’t access high-profile positions…. We have to choose between bringing up children, maternity and professional life. Men do not get involved in housework, nor in caring tasks…”* Key Informant 1.

Participants described the social expectations for young people and consequent punishment if the expectations are not met as being highly different for young women versus men. From the perspective of the interviewees, in the arena of sexuality, young women are expected to maintain their “reputation” while at the same time being sexually liberated: a balance which is impossible to achieve. The consequence is that young women are practically socially punished whatever they do; there is no way of avoiding criticism.

*“It seems that what this generation is seeking is something, it is freedom and… being very slutty in the bedroom, and so on…. But then, if you really are, they criticize you, because what remains is that a man, to be with a girl, she has to be valuable, hasn’t she? I think that it’s like a game. I don’t know. I think I told you earlier, the adjective is perverse, isn’t it? I think it’s perverse because it’s impossible to achieve.”* Key Informant 3.

*“They [girls] have to keep a balance between being sexy, but not too much; that is, between the dichotomy of virgin and whore. They are playing around this the whole time. They have to be very seductive so the boys like them, but at the right level, because if they exceed the limit then they are labelled a tramp, a whore, an easy girl, and they [girls] are wondering ‘Where do we have to place ourselves?’ We see this also…, the social pressure on young girls of not knowing what’s right and what’s wrong.”* Key Informant 4.

The social expectations for men continue to be those associated with what participants described as being rude, liking sports, dating as many girls as possible, and being violent, to cite a few characteristics. They are consequently discriminated against if they do not fit this ideal image of manhood. This discrimination for young men not matching these expectations was mentioned repeatedly during interviews, as was another unexpected issue: the rise of homophobic attitudes in young boys.

*“…and we have clearly also seen the issue of homophobia in the classrooms, the issue of having a determined model of masculinity.”* Key Informant 7.

These social norms were seen by interviewees as strongly rooted in society, and even as activists who are aware of these social constructions, it is sometimes difficult not to be influenced by them. The process of socialization through which these traditional gender roles are acquired begins from birth and continues throughout life, and it is very difficult, especially for young people, to question these if they do not have alternative models. This lack of models was pointed out during the interviews as being one of the main reasons why gender relations between young people have undergone relatively few changes, given the huge progress in formal equity.

*“How can we ask young generations to transform something when we are not giving them that information? I mean, we are telling them that simply because they are young, they have to solve this society’s problems and become equals and respect each other and so on; but we are not doing so, are we?”* Key Informant 3.

*“If they behave like that, it is because they replicate what they see; so, we cannot blame young people for things we haven’t changed in adults.”* Key Informant 10.

Mass media and cultural products were blamed by all the interviewees as producers of messages that support inequity, foster sexism, encourage violence and normalize IPV. In the words of a study participant, these “toxic messages” make it difficult to achieve equity. According to study participants, mass media – mainly TV advertisements, but also reality shows and soap operas – reinforce existing inequalities and traditional roles and models of femininity and masculinity. Cultural products consumed by young people, such as some popular musical styles, praise the subordination of women to men and even sometimes justify violence. Key informants provided numerous, detailed examples of popular films, books, and TV shows that display traditional roles for women and men and promote the idea of “romantic love” as the ideal to achieve.

*“Maybe it is enhanced by new offensives from patriarchy, from the music that is produced, that on the one hand it’s not anything new. I mean, songs and all the cultural industry are not creating new gender relation models; they are the same from the old scheme. But, well, in new TV series or films, they do reinforce the model of ‘romantic love’ and thus they maintain it, don’t they? And they generate idols where ‘macho’ continues being the example of the masculine and the passive, rescued woman is usually the example of the feminine…”* Key Informant 10.

At the same time, according to the participants, young girls feel that they live in an egalitarian society. The progress made towards equity in the last few decades in terms of laws, access to education and other basic rights for women may provide a false sense of equity to young girls. According to participants, equity is sometimes understood by young women as simply doing the same things that boys do, including not being respectful to their partner.

*“…that in effective relations there is much feeling of… ‘but I’m equal’. ‘If he controls me, I also control him’, ‘if he gets jealous, then I’m even more jealous’.”* Key Informant 3.

### IPV among young people: subtle, daily and normalized

When asked about IPV among young people, the interviewees described it as being closely linked to the characteristics of romantic relationships at this age. Young people’s intimate relationships were mainly described as being short-term, early in life, informal, with no well-defined boundaries, and more flexible than for adults.

For the interviewees the lack of boundaries and young age place young women in a vulnerable position, making them more permissive due to the uncertainty and fear of losing the relationship.

*“But at the same time as they engage in some relationships, they also establish many one-time relationships, don’t they? [They have] sexual relationships also at younger ages, and the younger you begin with those relationships, the less able you are to emotionally manage those relationships.”* Key Informant 4.

Interviewees also described young people’s relationships as strongly rooted in the idea of romantic love sold by the media and not questioning this model of relationship.

*“What we see at schools is even more centralized models, where they continue searching for their soulmate, their ‘Prince Charming’ that we all already know is the starting point of dependency relationships and quite dangerous.... We are discussing again ‘rape culture’, that is talked about so much now, and how we still blame the women in a situation. We also have to work, as my colleague was saying, towards the deconstruction of the model of masculinity and, of course, create other models that give an answer to diversity among individuals.”* Key Informant 7.

*“What they are less able to identify is that deconstruction; to challenge romantic love, street harassment, fear when going home – that’s what they identify less.”* Key Informant 3.

Although participants could recall particular cases or made reference to official reports of severe physical and sexual violence among this population, they described IPV among young people as subtle, normalized and daily, with the most frequent forms of violence being psychological abuse, and controlling social relations (deciding who the girl should and should not see) and dressing, for example.

*“Yes, I would say that it’s subtle. I would say that although there are cases of violence – let’s say physical, of course there is – but I would say that usually, what I find is mainly a lot of subtle violence, symbolic violence, of blackmail, of sibylline things that are not so easy to distinguish and identify….and, moreover, are quite frequent, although this is a personal interpretation.”* Key Informant 3.

In terms of controlling behaviour, participants talked about a modernization of IPV. Widespread use of new communication technologies by young people has changed some of the ways to exert control, however study participants believed that the underlying nature of this issue has not changed. Therefore, they believed that IPV was nowadays more dangerous in the sense that it can happen even when partners are not physically together, through threatening messages, for example, that can reach the receiver wherever she is, even in the privacy of her own home.

*“There are now new ways of harassment, which can be through new technologies and new ways. But the basis, I think, has remained stable from what it was previously.”* Key Informant 4.

*“Control through phone calls, control through WhatsApp, through social networks,… that’s quite current, but not because it did not happen in the past, but because it’s a new tool for communication among youngsters, and so violence goes on actualizing, modernizing. It gets adapted to modernity, it adapts to new tools used by young people. Nowadays it’s social networks and WhatsApp or the phone.”* Key Informant 4.

These characteristics – daily, normalized and subtle – along with the false feeling of equity described in the previous section make it difficult for young women to be aware of subtle and even explicit forms of violence, and react against them.

*“What happens in the moment, like not accepting ‘no’ for an answer, to understanding that some flirtatious remarks are not so, and those micro machismos that happen in adolescence, to identify and reject them, I think that that is what’s left.”* Key Informant 2.

*“That ‘he doesn’t allow me’ to meet with the girls is a recurring issue; “the thing is that he doesn’t allow me”, but who has to give you permission? Why does he have to allow you? That is an issue that surprises me, after so many years, but they happily say it many times. They like someone not allowing them and so on.”* Key Informant 9.

IPV was identified strongly during interviews as something common in young people. Participants supported their perception by referring to both existing official reports and also first-hand knowledge of individual cases. At the same time, they described some colleagues’ and politicians’ attitudes and reactions towards IPV among young people as denying this situation and declaring that equity has already been achieved, so neither further resources nor efforts should focus on this issue. One of the participants described controlling behaviour in young couples, such as controlling girls’ social relations, as frequent, but did not label it as IPV.

*“I have been working now for a lot of years with politicians and when you challenge the smallest thing about inequity between women and men, they range from those who deny it, – and that happens with technicians and civil servants as well – to those answering that a lot has already been done and so we shouldn’t complain so much because in other issues less has been done, to those who think we are exaggerating because equity has already been achieved, to those who feel really offended because we say that equity does not exist and they are committed to housework and taking care of their daughter. And then, these are people that continuously boycott you.”* Key Informant 6.

### Mass media campaigns do not fit young people’s needs

From our analysis of interview data, mass media prevention campaigns have, for a long time, sent messages that are far removed from young people. These messages have exclusively focused on physical violence, which study participants identified as an infrequent form of violence among young people, and have only targeted young adult women.

*“But I do have the feeling that, for example, campaigns which talk about maltreated women or violence against women… don’t reach… too much… young people’s reality.”* Key Informant 3.

According to the participants, prevention campaigns that exclusively show middle-aged women as disempowered victims of IPV make it increasingly difficult for young women, who perceive themselves as equal to men and empowered, to detect and recognize IPV in their own lives.

Interviewees described many successful local initiatives aimed at preventing IPV in youth implemented in previous years in the Basque Country. Perception of success of the described initiatives was built on participation rates and expressed satisfaction of target population in the absence of formal evaluation. However, only one of them had progressed from the pilot stage or small projects to a fully developed programme, due to a lack of political will and scarce resources assigned to addressing IPV.

*“There are plenty of things already planned to be done but there is no goodwill, so it’s a little bit exasperating. Then a new politician comes [along and wants to take action] because he/she has heard somewhere [about a project], and we say, yes, we know, we already did that, but as no human resources were allocated to continue the activity, it remained as a pilot project. Ah, they say, so do it again. No, I have no time to; you’ll have to hire some more people. There is no money for that. Thus it’s abandoned. And so on, all the time.”* Key Informant 6.

There was concern among the interviewees when reflecting on the messages of prevention campaigns, because there are no messages for men. Study participants believed the lack of messages for men is consistent with what happens in households; namely, that all of the messages are for girls to take care of themselves, but not for men to respect women.

*“What are we really transmitting there? I know it’s difficult, but many times I have discussed with my friends that with those messages of “Don’t go alone”, “Don’t wear that miniskirt”, “Don’t whatever”… why don’t we start giving our sons the same recommendations? ‘Listen Pepe, treat girls well, as equal to you. Don’t go around touching butts.’ Why? Because we do consider our daughter can be a target of rape and she is somehow tempting if she is coming home late or if she dresses provocatively, but we never consider that our son or whoever might [be a perpetrator], do we?”* Key Informant 7.

The participants identified the target population’s participation as the necessary key component for a successful intervention, from planning to programme implementation; however, this was seen as something difficult to achieve due to the huge distance between public institutions and scarce participation mechanisms for young people.

*“For me they (institutions) are really, really far. I mean the institutions are as far, far away from today’s youth as you can get.”* Key Informant 1.

This unsuitability of programmes does not mean the informants understood young women to be passive and just waiting to be “rescued” by public agencies from their vulnerability to IPV. At different points, all the interviewees made a claim vindicating young people’s agency. Key informants identified different coping strategies, some more successful than others, of young people despite this unfavourable environment for equitable relations. They identified emerging and promising different small initiatives which have been maintained by the personal commitment of young women towards more equitable gender relations.

*“Yes, we are able to see that there are young people willing to do things in another way… and there are young people that understand things in another way. …I think sometimes we demonize young people and, no, there are people, there are groups that do think things can be done differently and they do so.*” Key Informant 10.

“*I think that there is a part going backwards, but another one… Whether we want them to or not, young people do move. We cannot see everything from a negative perspective, because even if we do not take it into account, even if we don’t want to see it, there are people moving, there are young people moving and already making a change and, thus, what our programme wants is to optimize those strategies young people are already using in their daily life and also give them a value and make them a reference for other young people.”* Key Informant 3.

## Discussion

This study identified three themes that describe how participants characterized IPV among young people, and in turn how these characteristics, along with the wider context, affect the visibility of the problem and the characteristics of the existing programmes, as well as how mass media campaigns focused on this issue address it.

According to participants in this study, gender inequity was the basis of IPV. Although there is no agreement in the literature on the causes of IPV, recent research has found gender inequity at country level and normative gender roles to be related to IPV prevalence [28, 29]. Similarly, a research study conducted in Spain found that the risk of dying due to IPV varied among regions within the country and was related to scores on the Gender Development Index when comparing regions within the country [30]. Even at an individual level, support for gender equity has been identified as having protective effects against female IPV victimization and male IPV perpetration [31, 32].

Romantic and sexual relationships among young people were described by study participants as being different from those among adults; i.e., being shorter and having less defined boundaries and commitment. From their point of view, psychological abuse in the form of controlling behaviour is the most prevalent form of IPV which young girls are exposed to. This perception is supported by the results of the National Survey on Gender Violence, which found that as many as 19.4% of women aged 16–24 years reported exposure to this type of violence by current partners, while 1.5% reported physical violence by a current partner [10]. Psychological violence is the most difficult one to identify, not only for the girls exposed to it, but also for the adults responsible for policies and campaigns to address it [33].

Participants perceived that there is a gap between the huge strides towards equity in the formal sphere – i.e., legislation, access to the labour market, public discourse, etc. – and the slow changes in gender roles experienced in everyday life. This gap between the discourse about “how things should be” and practice has also been identified in research with young people in the Basque Country. Findings from these studies show that young people described more equitable relations in their discourses than they actually experienced in their own lives [34, 35]. Similarly, a large study on gender roles and stereotypes with Spanish adolescents found that only 21.8% of the participants questioned traditional gender roles and stereotypes. Moreover, half of the participants strongly supported traditional views about couples, such as the girl being submissive to her partner’s desires [36]. Other studies have also found that despite changes in recent decades, girls and boys continue to be socialized differently, following traditional gender roles especially regarding the concept of love [37]. The scarcity of models that challenge traditional norms might to some extent explain this gap. Alternate models of gender equity do have a positive impact on the development of young people’s gender roles, as shown by research done in the Basque Country; however, having such alternate models in their proximity was not commonplace for participants in these studies [34, 35]. In this regard, the mass media play a key role in the normalization of violent behaviours through the sustainment of “romantic love” as the unique model for romantic relationships in the view of the participants, a perception supported by findings from research on the effect of the media on gender construction [37, 38]. The model of “romantic love” has been lately described in many studies as being supportive of unequal relations based upon traditional roles for women and men and consequently increasing vulnerability to and normalization of IPV [37, 39]. Moreover, the mass media contribute to the invisibility of IPV, minimizing its relevance and questioning whether it is something real or something women make up [40].

The social cognitive theory of gender development [41] is a useful framework for further analysing the significance of models and mass media in the development of gender roles. According to this theory, gender roles are shaped as a result of the flexible and changing interactions between three influences: modelling, enactive experiences, and direct tuition. Modelling refers to the process of abstracting the rules and structures underlying the (in this case) gender-linked behaviour of significant others (mainly family, peers and media) and putting it into practice. Enactive experience relates to learning through social sanctions or approval received after enacting a gender-linked behaviour. Finally, direct tuition refers to explicit messages about what gender-linked behaviour should be like. In this theory, the influence exerted by modelling and enactive experiences is much stronger than direct tuition. In addition to the scarcity of models that promote equity, especially in the mass media, participants described strong social sanctions for girls who do not follow the gender-linked behaviour expected of them. Other authors have also described the strong pressure society puts on young women to meet traditional gender roles and the negative consequences this has for equity and IPV prevalence [37]. In this regard, it seems difficult to envisage that the implementation of prevention strategies can counterbalance, through the influence of direct tuition, the enormous effect that modelling and enacted experiences have on maintaining inequity. This might explain the findings of Franco [17] where, despite all the efforts expended in Spain on addressing IPV among young people during the past decade, rates of victimization remain similar and tolerance towards IPV has increased.

The prevention strategies identified by participants in this study can be broadly divided into two groups: nationwide mass media campaigns and locally implemented school-based projects. Traditionally, mass media campaigns and messages aimed at preventing violence have mainly been focused on adults and physical violence, far removed from the typical characteristics of IPV among young people in the view of the participants. The messages target women exposed to violence, and encourage them to get out of the violent situation. Only one of the campaigns launched by the Spanish Health, Social Services and Equity Ministry during the past 10 years explicitly encouraged society to challenge perpetrators [42]. According to the participants’ perception, the stereotype of IPV portrayed in these mass media campaigns has been middle-aged white women exposed to physical violence. The positive effect of these campaigns has been to bring IPV into the public sphere and raise awareness about the problem. However, as explained before, physical violence is not the most prevalent type of IPV among youth, and violence usually begins at a young age. More recent campaigns have tried to address this limitation by shifting the focus to young women exposed to psychological abuse, but they still maintain the emphasis on encouraging women to leave the violent relationship as the solution to IPV [42].

According to the literature, school-based IPV prevention programmes can have a positive impact on changing violence-supporting attitudes, increasing knowledge, and reducing its prevalence [19, 43, 44]. School-based projects implemented by participants incorporated some of the recommendations found in the literature for successful prevention of IPV, such as including a significant skill-building component, addressing myths and stereotypes relating to IPV [20], and ensuring flexibility, fidelity and sensitivity to diversity [45]. Consistent with participants’ views of young people’s agency, these local initiatives put youngsters’ participation at the core of the interventions. Therefore, locally implemented programmes ranged from a short film contest to give a voice to young people about how they understand IPV and its prevention to a peer education programme fully led by secondary school students with the support of equity technicians. However, all of these local initiatives failed to include a rigorous outcome evaluation to demonstrate the effectiveness of the implemented interventions and, thus, failed in producing tools to advocate to politicians for their sustainability as recommended in the literature [20].

Although participants described some school-based interventions as successful, it is worth pointing out that these interventions focused on the top tiers of the Health Impact Pyramid developed by Frieden [46]; i.e., at changing individual behaviour. As the author pointed out, these interventions, if universally and effectively applied, can have an impact on the population, but changes are difficult to maintain if the context is not supportive for them [46, 47]. To effectively prevent IPV, it is therefore essential to first ensure that successful interventions are universally and effectively applied. To achieve this objective, implemented interventions need to address their evaluation deficiency by including experimental outcome evaluations, including the measurement of changes in behaviour in addition to knowledge and attitudes. Secondly, there is a need to change the broader socio-cultural context by challenging mass media messages that support and foster inequity, and offering new models of healthy romantic relationships and of gender-linked behaviour that breach traditional gender roles. This second objective is difficult to achieve with only the commitment of frontline professionals and instead requires strong political commitment.

### Methodological considerations

We applied the criteria described by Lincoln and Guba [48] to enhance credibility in qualitative research. Credibility was enhanced by prolonged engagement, as the first author (AM) grew up, lives, and works in the research setting. To counterbalance the naivety of prolonged engagement, we used a triangulation of researchers, with the other three authors being foreign to the research setting. To stay closer to the text, the original Spanish version was used in the coding process and only when the themes had emerged did translation into English take place. We made an effort to include all the sectors involved in our topic of interest: IPV and young people in the Basque Country. It would have been interesting to incorporate the perspectives of those at a decision-making or political level, but although some politicians were invited to participate in the study, for different reasons (lack of time, suggesting interviews with technical staff instead, or lack of response) they eventually did not do so.

We have tried to describe the setting and context in order to enable readers to assess the applicability of our results to other places and circumstances. In this regard, it is important to highlight the historically longer commitment of the Basque Government to gender equity compared with other regions in Spain, which is reflected in specific legislation and the power of the local Institute for Women’s Affairs, Emakunde.

A possible limitation of this study was the difficulty in getting potential participants to agree to be interviewed. Those who decided to take part might have been driven by a personal commitment to the issue under study and so differ in their perspectives from others who did not respond to the invitation. Including professionals from the four sectors most relevant to our research question results in a more comprehensive perspective on the topic, but might be a limitation in the sense that is difficult to assess whether saturation was reached in all the sectors.

To enhance confirmability, we followed the steps proposed by Braun and Clarke [27], going back to the interviews many times to check for consistency between the themes developed and the data. With this same aim, many quotations have been included in the results, allowing the readers to judge the interpretations made. This study reflects only the perceptions of the professionals interviewed, which might provide a partial picture. Future studies that are part of this research project will address this limitation, comparing these findings with young people’s perceptions.

Finally, in order to increase dependability, we followed an emerging design, responding to new issues raised by interviewees and including these in later interviews e.g. the role the mass media plays in the construction of equity between women and men.

## Conclusions

The study participants showed good knowledge of the characteristics of IPV among young people. They identified psychological abuse in the form of controlling behaviour by partners as the most common type of IPV young women are exposed to, although they were also aware of the exposure to other types of IPV. This violence was described as something subtle, daily and normalized. As a result, it was not easy to recognize by the girls being exposed to it, or even by the adults working with young people.

Their knowledge of IPV was reflected in implemented local IPV prevention projects, which study participants considered successful in addressing young people’s needs. However, these interventions lacked formal evaluation, political support, and continuation.

The study participants did not believe that nationwide mass media campaigns realistically reflected the specific characteristics of IPV among young people. Thus, participants perceived these campaigns to be ineffective.
